# Androgen Receptor Signaling and the Emergence of Lethal Neuroendocrine Prostate Cancer With the Treatment-Induced Suppression of the Androgen Receptor: A Literature Review

**DOI:** 10.7759/cureus.13402

**Published:** 2021-02-17

**Authors:** Meera Dhavale, Mohamed K. Abdelaal, A B M Nasibul Alam, Tatjana Blazin, Linha M Mohammed, Dhruvil Prajapati, Natalia P Ballestas, Jihan A Mostafa

**Affiliations:** 1 Research, California Institute of Behavioral Neurosciences & Psychology, Fairfield, USA; 2 Internal Medicine, California Institute of Behavioral Neurosciences & Psychology, Fairfield, USA; 3 Psychiatry, California Institute of Behavioral Neurosciences & Psychology, Fairfield, USA

**Keywords:** neuroendocrine carcinoma of prostate, androgen receptor, castration resistant prostate cancer, androgen deprivation therapy, prostate cancer, anti-androgen therapy, treatment emergent neuroendocrine prostate cancer, neuroendocrine differentiation, advanced prostate cancer, aurora kinase inhibitor

## Abstract

Androgen receptor signaling primarily influences both the normal growth and proliferation of the prostate gland and the development of prostatic carcinoma. While localized prostate cancers are typically managed with definitive therapies like surgery and radiotherapy, many patients have recurrences in the form of metastatic disease. Androgen deprivation therapy, by way of castration via orchiectomy or with drugs like luteinizing hormone-releasing hormone (commonly called gonadotropin-releasing hormone) agonists and luteinizing hormone-releasing hormone antagonists, is the primary mode of therapy for advanced castration-sensitive prostate cancer. Castration resistance invariably develops in these patients. Further treatment has shifted to newer anti-androgen drugs like enzalutamide or abiraterone and taxane-based chemotherapy. Prolonged inhibition of the androgen receptor signaling pathway causes androgen receptor-independent clonal evolution which leads to the development of treatment-emergent neuroendocrine prostate cancer.

All prostate cancers at the initial presentation should undergo evaluation for the markers of neuroendocrine differentiation. Detection of serum biomarkers of neuroendocrine differentiation and circulating tumor cells is a prospective non-invasive method of detecting neuroendocrine transdifferentiation in patients undergoing treatment with androgen receptor pathway inhibitors. It is essential to perform a biopsy in the presence of red flags of neuroendocrine differentiation. Alisertib, an Aurora kinase inhibitor, showed promising clinical benefit in a subgroup of patients with certain molecular alterations. A thorough understanding of the molecular and clinical programming of treatment-emergent neuroendocrine prostate cancer can potentially lead to the development of drugs to prevent the development of this lethal variant of prostate cancer.

## Introduction and background

Prostate cancer is the most common cancer aside from skin cancers and the second leading cause of cancer-related death in men in the United States. About 191,930 new cases and 33,330 prostate cancer-related deaths were expected to occur in 2020 [1]. It typically begins as prostatic intraepithelial neoplasia (PIN), which transforms into localized prostate cancer. The localized prostate cancer may then transform into locally invasive advanced adenocarcinoma, leading up to metastatic prostate cancer. The aggressiveness of prostate cancer is best defined by the Gleason grading system, which grades the tumors based on histological patterns of prostatic adenocarcinoma [2].

Localized prostate cancers are primarily treated with definitive therapies, like surgery and radiotherapy. Despite the effectiveness of these therapies, 30% of patients have recurrences in the form of metastatic disease, with the five-year survival being only 29% in such cases [3]. 

Ever since Huggins and Hodges first demonstrated the efficacy of the technique to treat metastatic prostate cancers in 1941, androgen deprivation therapy (ADT) in the form of castration via orchiectomy or using luteinizing hormone-releasing hormone agonists (LHRH agonists) and luteinizing hormone-releasing hormone antagonists (LHRH antagonists) has been the first line of management for advanced prostate cancers [4]. ADT is also sometimes used as a neoadjuvant/adjuvant therapy with radiation [4]. The goal of androgen deprivation is to reach castration levels of testosterone (<50 ng/dL; <1.7 nmol/L), which is associated with improved cause-specific survival [5]. 

Despite primary treatment with ADT, some patients experience recurrences. These castration-resistant prostate cancers (CRPC) are usually correlated with rising prostate-specific antigen (PSA) levels, which is indicative of androgen receptor-driven activity [6]. At the moment, newer anti-androgen drugs like enzalutamide and abiraterone, and/or taxane-based chemotherapy [7,8], are used to manage CRPCs. 

Substantial evidence now supports the correlation between the development of CRPC and continued transactivation of the androgen receptor [6,9]. Common mechanisms of castration resistance include alterations in the androgen receptor-signaling pathway, androgen receptor-signaling bypass mechanisms, and androgen receptor-independent clonal evolution. The latter mechanism is thought to cause the lethal form of CRPC called treatment-emergent neuroendocrine prostate cancer (t-NEPC) [10]. t-NEPC incidence rates are increasing with the widespread use of potent androgen receptor pathway inhibitors [6]. Table 1* *summarizes the results of five research studies that chronicle the occurrences of neuroendocrine prostate cancer in patients who have undergone some form of androgen deprivation therapy.

**Table 1:** A summary of research articles that chronicle the occurrences of treatment-emergent neuroendocrine prostate cancer. ADT: Androgen Deprivation Therapy; PSA: Prostate-Specific Antigen; NEPC: Neuroendocrine Prostate Cancer; t-NEPC: treatment-emergent NEPC; NED: Neuroendocrine Differentiation; CRPC: Castration-Resistant Prostate Cancer; CGA: Chromogranin A.

Author	Study design	Study subjects	Results
Beltran H et al. [11]	Case series	Three patients	All patients who had previously been treated with ADT presented with low serum PSA levels. Their clinical pictures corresponded to the transformation of prostatic adenocarcinoma to t-NEPC.
Ito T, et al. [12]	Retrospective study	137 whole prostate specimens	NED markers were detected in 70.5% of patients who underwent ADT for >13 months compared to 30% in those that hadn’t received any ADT.
Aggarwal R, et al. [13]	Multi-institutional prospective study	202 patients	The study concluded that t-NEPC develops in almost one out of 5 individuals (~17%) with CRPC previously treated with ADT.
Hirano D, et al. [14]	Retrospective study	93 prostate cancer specimens	49/93 (53%) tumors tested positive for CGA. The NED status seemed to rise with longer durations of treatment with ADT (p <0.0001).
Conteduca V, et al. [15]	Prospective study	87 NEPC specimens	47 (54%) NEPC cases presented de novo, & 40 (46%) were t-NEPC. The median time from adenocarcinoma to t-NEPC diagnosis was 39.7 months with an average of 2 antecedent systemic therapies.

A delay in diagnosis and lack of specific therapies make t-NEPC a lethal form of prostate cancer with a mean survival of seven months [16]. A thorough understanding of the role of androgen receptor signaling is important as it plays a critical part in the normal growth and development of the prostate gland as well as in prostate carcinogenesis and its progression into castration-resistant cancer and neuroendocrine prostate cancer [9]. In this article, we review the physiology of androgen receptor signaling, studies that present different mechanisms of neuroendocrine transformation of prostate cancer, and potential neuroendocrine prostate cancer (NEPC)-specific markers for targeted management.

## Review

Androgen receptor and its signaling pathway

**Figure 1:**
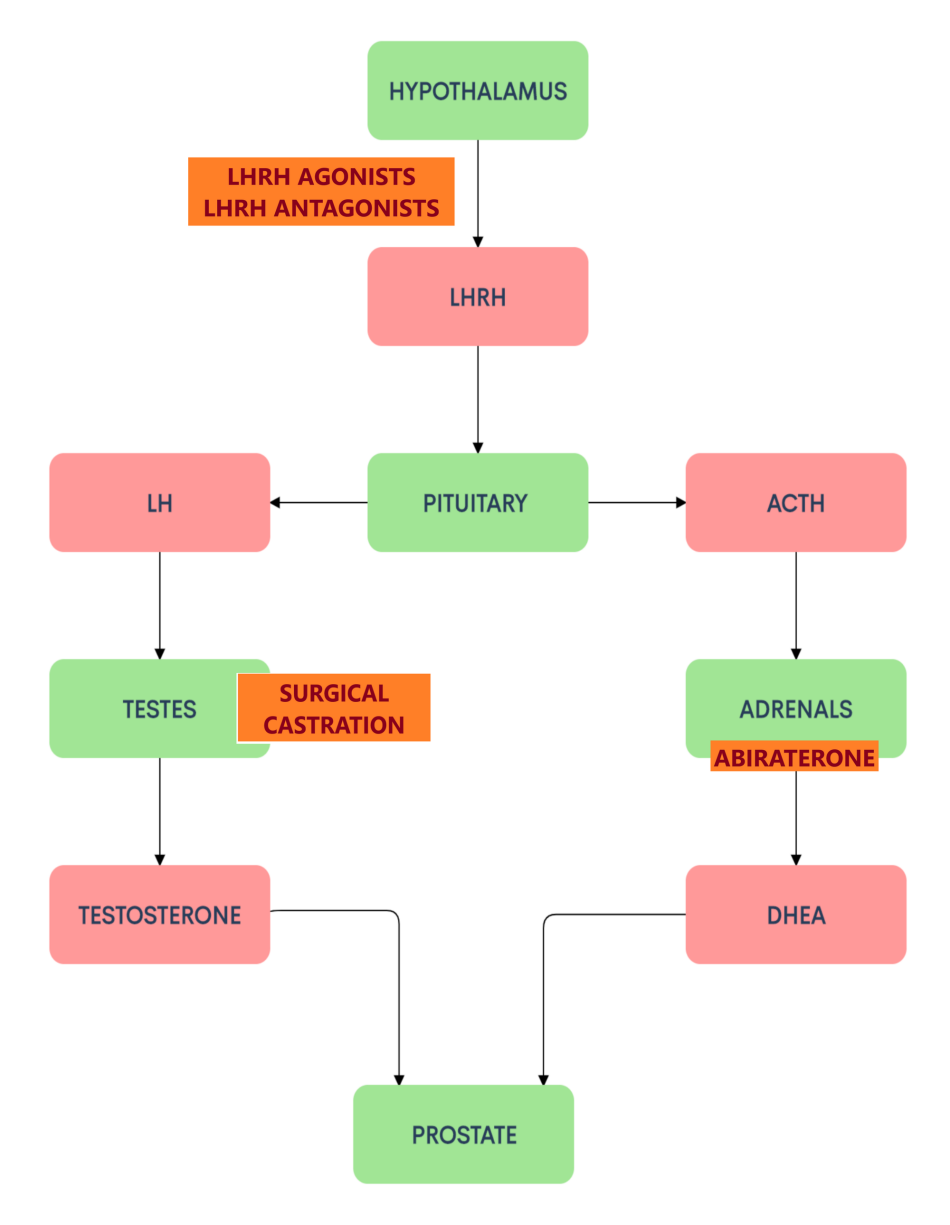
Central regulation of androgen production and the sites of action of ADT and abiraterone. Androgen Deprivation Therapy (ADT) comprises castration by way of surgery (orchiectomy) or using drugs like LHRH agonists and LHRH antagonists [4]. Abiraterone is an antiandrogen drug that inhibits the production of androgens [19]. Both these therapies are commonly used in the treatment of prostate cancers. LHRH: Luteinizing hormone-releasing hormone; LH: Luteinizing hormone; ACTH: Adrenocorticotropic hormone; DHEA: Dehydroepiandrosterone.

Androgens like testosterone and dihydrotestosterone (DHT) are indispensable for the development of the male reproductive system and secondary sexual characteristics [17]. Androgens in adult males are primarily produced by the testes. The production of these gonadal androgens is influenced centrally by the hypothalamus and the anterior pituitary. Pulsatile secretion of LHRH from the hypothalamus drives the expression of luteinizing hormone (LH) from the anterior pituitary, which in turn stimulates the interstitial Leydig cells in the testes to produce testosterone [18]. Figure 1 describes the central regulation of androgen production.

Testosterone and dihydrotestosterone are steroidal hormones that exert their effects through a ligand-dependent nuclear transcription factor present in the cytoplasm, called the androgen receptor (AR). The enzyme 5-alpha-reductase converts testosterone to dihydrotestosterone (DHT), which is a more potent agonist of the androgen receptor [20].

**Figure 2:**
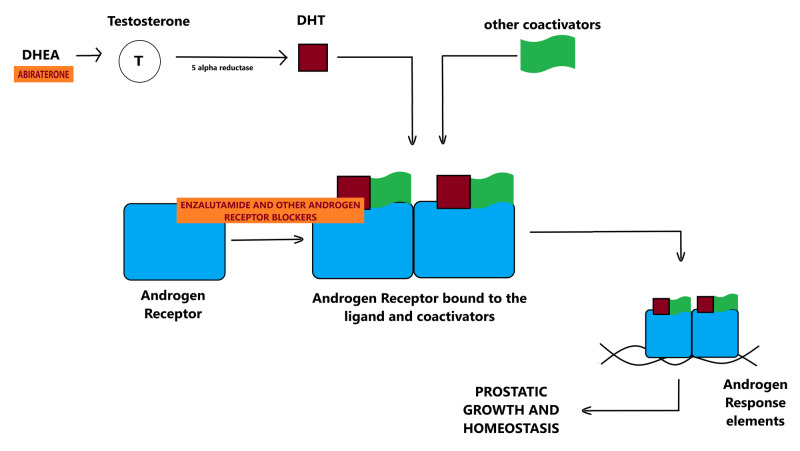
Androgen receptor signaling and sites of action of abiraterone and enzalutamide. Enzalutamide is an androgen receptor blocker used conjointly with androgen deprivation therapy in the treatment of prostate cancers [19]. DHEA: Dehydroepiandrosterone; DHT: Dihydrotestosterone.

The AR has three functional regions: the ligand-binding domain, the DNA binding domain, and the N-terminal domain. Endogenous androgens (like DHT) activate the receptor by attaching to its ligand-binding domain in the cytoplasm. This is followed by a conformational change in the receptor which undergoes hyperphosphorylation along with the release of heat shock proteins. The activated androgen receptor then translocates to the nucleus where the AR-ligand complex undergoes dimerization, engages with other co-activators, and undergoes DNA binding. The androgen receptor has zinc fingers that act as hormone-recognition structures for specific androgen response elements (ARE). This enables direct tethering to the promoter region of target genes [17]. Figure 2 illustrates the androgen receptor signaling and indicates the sites of action of some androgen receptor pathway inhibitors.

When a patient undergoes androgen deprivation in the form of either medical or surgical castration, the production of gonadal androgens ceases. The adrenal glands continue secreting weaker androgens like dehydroepiandrosterone (DHEA) and androstenedione. These weaker androgens supply precursors that are converted to more potent forms of androgens, like testosterone and DHT in the periphery, which go on to activate the androgen receptor. This is thought to be a mechanism of the development of CRPC [21,22]. 

Most patients with CRPC continue to show evidence of androgen receptor-driven activity indicated by progressive cancer with a rising PSA level [6]. Hence studies recommend combining ADT with other classes of drugs in the initial treatment of advanced castration-sensitive prostate cancers (CSPC). For this reason, newer anti-androgen drugs like abiraterone (inhibits the synthesis of testosterone) and apalutamide/enzalutamide (androgen receptor antagonists), or docetaxel (taxane-based chemotherapy), are used conjointly with ADT [22]. 

Neuroendocrine prostate cancer

While newer and more potent anti-androgen medications like enzalutamide and abiraterone are correlated with longer survival in patients with CRPC, resistance invariably develops to these therapies as well [7,8]. This occurs most commonly through alterations in the androgen receptor-signaling pathway, androgen receptor-signaling bypass mechanisms, or androgen receptor-independent clonal evolution.

Androgen receptor-independent clonal evolution gives rise to treatment-emergent neuroendocrine prostate cancer (t-NEPC) [6]. The median length of survival among patients with t-NEPC is seven months [16]. De novo small cell carcinoma of the prostate is extremely rare and resembles small cell cancers of the lung. In comparison, t-NEPC can have a pure small cell morphology or a mixed morphology since they evolve from prostatic adenocarcinoma as a mechanism of resistance [6,23].

Clinical Presentation of Neuroendocrine Prostate Cancers

Aparicio et al. [24] empirically described the following seven clinical features as characteristic of neuroendocrine prostate cancer based on their experience and evidence presented by previous work [25-30]: progression to androgen-independent tumors in a short interval after androgen deprivation therapy; bulky high-grade tumor mass in the prostate or pelvis or bulky lymph nodes; histopathological confirmation of small-cell prostate cancer; low PSA at initial presentation of castration naive prostate cancer or after ADT, with abundant osseous metastases; positive staining of neuroendocrine markers like chromogranin A or synaptophysin on histological examination or elevated levels of chromogranin A in the blood in addition to the unwarranted elevation of calcium, LDH (lactate dehydrogenase), or CEA (carcinoembryonic antigen) levels in the serum; only visceral metastases; and predominant lytic osseous metastases on X-Ray or CAT scan.

The variegated clinical picture of neuroendocrine prostate cancers makes them elusive at presentation. They usually present with a combination of aggressive metastatic disease and low serum PSA levels. A biopsy and histopathological review are necessary to definitively diagnose NEPC and its subtypes. These cancers present with aggressive metastatic disease and biopsies of metastatic lesions are generally avoided unless neuroendocrine differentiation is suspected. The absence of markers like PSA and AR, in combination with the presence of markers of neuroendocrine differentiation (NED) like chromogranin A (CGA), neuron-specific enolase (NSE), and synaptophysin (SYP), aids the diagnosis of neuroendocrine prostate cancer [11,31]. A delay in diagnosis and limited treatment options make neuroendocrine prostate cancer a lethal subtype of castration-resistant prostate cancer [6]. 

Mechanisms of Neuroendocrine Differentiation

This paper presents a review of prior work on neuroendocrine differentiation. Following is a brief account of their aims and results:

Molecular model of neuroendocrine prostate cancer progression: Chen et al. argue that cooperation between multiple presumptive molecular drivers and facilitators is implicated in the development and progression of neuroendocrine prostate cancer [6]. According to the model presented in the paper, genes like N-Myc, RB1, and TP53 aid evasion from androgen deprivation therapy in an androgen receptor-independent manner through lineage plasticity; genes like SRRM4, REST, BRN2, and FOXA1/2 play a crucial role in specific neuroendocrine differentiation of the tumor; and mediators like AURKA, PEG10, MEAF6, and Cyclin D1 allow neuroendocrine prostate cancer cells to undergo clonal expansion and develop treatment resistance.

Cancer cell fusion and androgen-independent progression: Yin et al. studied the interaction between prostate cancer cells and neural cells using three-dimensional co-cultures of neurospheres and androgen-sensitive human prostatic adenocarcinoma cells. Upon induced neural differentiation, the results indicated the fusion of cancer and neural stem cells, which survived longer in a latent state. Clones of these hybrid lineages showed heterogeneity, had lost their prostatic and epithelial markers, and some had gained neural markers suggesting cancer cell and neural cell fusion in the tumor microenvironment. Random samples of these hybrid lineages showed no or subdued sensitivity to androgen stimulation, suggesting androgen receptor-independent clonal evolution [32].

Role of AURKA and MYCN (genes that code for Aurora kinase A and n-Myc respectively): Beltran et al. profiled tissue samples from neuroendocrine prostate cancer (NEPC), prostatic adenocarcinoma, and benign prostatic tissues, through ribonucleic acid (RNA) sequencing and oligonucleotide arrays using immunochemistry and fluorescence in situ hybridization (FISH). AURKA and MYCN were overexpressed in over a third of NEPC signifying they cooperate to induce neuroendocrine differentiation. Both genes were also overexpressed in local prostate cancer tissue samples, possibly indicating an at-risk group. NEPC cells responded noticeably to the administration of Aurora kinase inhibitor therapy both in vivo and in vitro, followed by the complete suppression of neuroendocrine markers. The results of the study support the potential role of Aurora kinase inhibitors in the treatment of neuroendocrine prostate cancers with these genetic alterations [33].

Role of transcription factor-4: A study conducted by Lee et al. tried to determine the mechanism of resistance of castrate-resistant prostate cancer to enzalutamide. RNA sequence analysis and examination of tissues obtained from men who had died from metastatic prostate cancer showed an association between increased levels of transcription factor-4 (TCF-4) and increased levels of neuroendocrine markers in the prostatic adenocarcinoma cell line resistant to enzalutamide when compared to the parental cell line. The results of the study stipulated that TCF-4 influences enzalutamide resistance through neuroendocrine differentiation [34].

Role of neurotensin and its receptors: A potential strategy to avoid the onset of neuroendocrine differentiation was suggested by Zhu et al. Their study argued that androgen deprivation generates neurotensin in cell lines and animal models of castrate-resistant prostate cancers with neuroendocrine features. They found that neurotensin causes neuroendocrine differentiation by the activation of neurotensin receptor 1 (NTSR1) and neurotensin receptor 3 (NTSR3), not neurotensin receptor 2 (NTSR2). Their findings indicated that inhibition of the NTSR1 signaling pathway prevented the development of neuroendocrine differentiation and castration resistance in vivo, suggesting a potential preventive strategy that can avert the onset of neuroendocrine differentiation [35].

Beta-adrenergic signaling: Sang et al. hypothesized that G protein-coupled receptor kinase 3 (GRK3) induces neuroendocrine differentiation of prostate cancer. Beta-adrenergic signaling activation causes cyclic adenosine monophosphate (cAMP) response element-binding (CREB) protein pathway activation which has been implicated in promoting angiogenesis and prostate cancer progression. Their study demonstrated that CREB activation caused an increased expression of the GRK3 and neuroendocrine markers in prostate cancers previously managed with ADT. Treatment with isoproterenol (a beta-adrenergic agonist) stimulated this pathway and propranolol (a beta-adrenergic blocker) averted the NED [36].

Leukemia inhibitory factor (LIF): The study conducted by Liu et al. argues that LIF is a formidable serum biomarker of advanced prostate cancer. This study demonstrated that activation of LIF signal transduction and STAT3 signaling promotes neuroendocrine differentiation of prostate cancer treated with androgen deprivation therapy. This mechanism is arbitrated by castration-induced ZBTB46 (a transcription factor that is pivotal for prostate cancer metastasis) and inhibiting the ZBTB46-LIF pathway could potentially inhibit the development of castration resistance and neuroendocrine differentiation after medical or surgical castration [37]. 

Management of neuroendocrine prostate cancer

Workup 

While histopathological evaluation is performed at the initial presentation of prostate cancer, patients don’t typically undergo evaluation for neuroendocrine prostate cancer. Newer studies recommend performing a workup for neuroendocrine prostate cancer at the initial presentation itself, by looking for neuroendocrine markers and molecular alterations implicated in the process of neuroendocrine differentiation of prostate cancers. Such a workup would rule out de novo neuroendocrine prostate cancer and aid in identifying high-risk groups [38]. Nevertheless, these recommendations are based on preclinical evidence and further research is warranted.

When NEPC presents with visceral and/or osseous metastases, biopsies of these metastatic lesions are avoided due to the belief that the risks related to the invasive procedures outweigh the benefits. However, a histopathological assessment of these metastatic tissue samples would facilitate early diagnoses. Ineffective treatments targeting the androgen receptor signaling pathway can be avoided, thereby eliminating the chance for adverse effects from the drugs and reducing treatment costs. An early diagnosis would also allow timely administration of efficacious chemotherapy to curb disease progression.

A non-invasive way of detecting t-NEPC is to identify serum biomarkers of NED. Markers like neuron-specific enolase (NSE), chromogranin A (CGA), and synaptophysin (SYP) are already being used in the histological examination of neuroendocrine prostate cancer [38]. They can be easily detected in the serum of patients with prostate cancer using techniques like enzyme-linked immunosorbent assay (ELISA). There are reports that provide evidence that CGA and NSE are upregulated in patients who have undergone hormonal therapy [39], and higher levels before or during abiraterone treatment are predictors of poorer survival [40,41]. Other non-invasive markers to detect neuroendocrine prostate cancer include circulating neuroendocrine tumor cells in the serum. Beltran et al. demonstrated that the circulating tumor cells from patients with neuroendocrine prostate cancer exhibited a unique structure, had subdued androgen receptor expression, and lower epithelial markers compared to those from patients with castration-resistant prostate cancer [42]. While further research is needed to corroborate these findings, these are promising results. 

Treatment

There hasn’t been any recent advancement in the guidelines for the treatment of neuroendocrine prostate cancer despite numerous research studies being conducted. The current recommendations advise treating them similar to anaplastic castrate-resistant prostate cancer as they have analogous clinical features. The first line of treatment for the management of localized or metastatic advanced prostate cancers is platinum-based chemotherapy which is a combination of cisplatin/carboplatin with etoposide or docetaxel [24]. While there is a high response rate to these chemotherapy regimens, they are associated with notable toxicities and don’t cause long-term remission [43].

Pre-clinical studies and clinical trials are being conducted for targeted therapies for t-NEPC. A Phase II clinical trial of alisertib, an AURKA inhibitor, demonstrated significant clinical benefit in a subset of patients with AURKA and N-Myc activation [44], despite not meeting its primary endpoint. Programmed death-ligand 1 (PD-L1) inhibitors (avelumab) and Delta-like ligand 3 (DLL3) inhibitors (rovalpituzumab) are other drugs currently being studied for the treatment of NEPC [45,46]. Both PD-L1 and DLL3 inhibitors have proven useful in the treatment of small-cell lung cancers, which resemble NEPCs [47].

Clermont et al. [38] have proposed the following set of clinical guidelines that coalesces the clinical and molecular features of neuroendocrine prostate cancer. They suggest that the initial histopathological examination should also include markers of neuroendocrine differentiation (like CGA, NSE, and SYP) to rule out de novo NEPC. Advancements in research to detect sensitive molecular alterations at this stage could help predict treatment-emergent NEPC. The periodic monitoring of serum markers (like chromogranin A, neuron-specific enolase), circulating tumor cells, and circulating tumor DNA/RNA could aid in detecting the development of transdifferentiation or treatment resistance. However, further research is needed to determine their sensitivity and specificities to design better non-invasive tests. Biopsies should be promptly performed in the presence of red flags of NED like rapidly progressive lesions with disproportionate PSA levels, visceral metastases, paraneoplastic syndromes, or positive blood work findings mentioned above. As per the current standard treatment, the first line of management on confirmation of t-NEPC should be cisplatin-etoposide chemotherapy. The use of Aurora kinase A inhibitor alisertib as targeted therapy is supported by multiple clinical studies and has the potential to be included in the standard management of NEPC in the near future.

Limitations

As a traditional review (not a systematic review), this article didn’t follow any specific protocol for data collection, and the papers included weren’t subjected to quality assessment. These limitations can be a cause of researcher bias. Many studies reviewed in this article presented pre-clinical evidence, and therefore these claims presented require further investigation.

## Conclusions

The adoption of newer and more potent anti-androgen medications like enzalutamide and abiraterone is expected to result in an increase in t-NEPC incidence. The diverse presentation of NEPC causes a delay in diagnosis, while limited treatment options make it lethal. Since a histopathological examination can definitively diagnose the presence of neuroendocrine differentiation, we suggest that all prostate cancers undergo evaluation for markers of NED at the initial presentation. Pre-clinical evidence suggests periodic monitoring of serum biomarkers and circulating tumor cells could aid in detecting transdifferentiation of prostatic adenocarcinoma into NEPC. It is imperative to perform a biopsy in the presence of red flags of NED.

This review paper recapitulates the role of androgen receptor signaling in the development of NEPC. It is important for physicians to look out for NED in patients with prostate cancer, especially in those who have undergone ADT, in order to start specific therapy early in the disease progression. Apart from taxane-based chemotherapy, alisertib, an AURKA inhibitor, has demonstrated the potential to be a targeted therapy in patients with certain molecular alterations. More clinical trials and research studies need to be conducted for the development of non-invasive diagnostic tests and other targeted therapies. A thorough understanding of the molecular and clinical programming of t-NEPC can potentially lead to the development of drugs that prevent the development of this lethal variant of prostate cancer.
